# Association between consumption of dairy products and incident type 2 diabetes—insights from the European Prospective Investigation into Cancer study

**DOI:** 10.1093/nutrit/nuv018

**Published:** 2015-07-11

**Authors:** Nita G. Forouhi

**Affiliations:** *N.G. Forouhi* is with the Medical Research Council Epidemiology Unit, University of Cambridge School of Clinical Medicine, Institute of Metabolic Science, Cambridge Biomedical Campus, Cambridge, UK.

**Keywords:** dairy, EPIC study, saturated fatty acids, type 2 diabetes, yogurt.

## Abstract

The public health burden of type 2 diabetes has risen unabated over the past decades, fueled by obesity and lifestyle influences, including diet quality. Epidemiological evidence is accumulating for an inverse association between dairy product intake and type 2 diabetes risk; this is somewhat counterintuitive to the saturated fat and cardiometabolic disease paradigm. The present report reviews the contribution that the findings of the European Prospective Investigation into Cancer (EPIC) study have made to this debate, noting that types of dairy products, particularly fermented dairy products including yogurt, may be more relevant than overall dairy intake for the prevention of type 2 diabetes. The EPIC study has contributed evidence through complementary approaches of a large prospective study across 8 European countries with heterogeneous dietary intakes assessed using food-frequency questionnaires (EPIC-InterAct study) and through a more detailed examination of diet assessed using a 7-day food diary (EPIC-Norfolk study). The implications of these findings are placed in the wider context, including the use of individual fatty acid blood biomarkers in the EPIC-InterAct study and an appraisal of current research gaps and suggestions for future research directions.

## INTRODUCTION

The global burden of diabetes mellitus is high, and increasing, with the latest estimates from the International Diabetes Federation suggesting that 382 million people had diabetes in 2013; that number is projected to increase to 592 million by 2035.^1^ The multiple serious consequences of diabetes, including macrovascular and microvascular complications that lead to premature morbidity and mortality, pose a major threat to public health. For type 2 diabetes, the most common form of diabetes, there is high-quality evidence from clinical trials in diverse settings that lifestyle interventions are effective for it's primary prevention.^2–6^ However, in day-to-day practice in real-world settings outside of clinical trials, uncertainty remains about the specific dietary factors that relate to diabetes risk and the optimal dietary advice for individuals and populations.

There is increasing interest in the potential role that dairy products might play in diabetes etiology, though research evidence has been mixed as to whether different types of dairy products have a beneficial, detrimental, or null association with type 2 diabetes.^7–10^ The focus within dietary guidelines to reduce the consumption of saturated fat for the prevention of cardiovascular disease^11^ has generally supported the view that dairy products should be consumed for bone health. However, as they are typically high in saturated fat content, dairy products should be consumed in moderate amounts and as low-fat varieties. A specific example of caution against dairy products is the “reverse coding” within algorithms for estimating adherence to a Mediterranean diet pattern, whereby dairy foods are given a “reverse” coding with a score of 0 for high (at or above median) and 1 for low (below median) dairy product consumption, while the converse is the case for perceived “healthy” foods such as fruits, vegetables, legumes, cereals, and fish.^12^ In a previous study, a similar principle of assigning a value of 0, 1, or 2 to intakes of first, second, and third tertiles of intakes of the “beneficial” components of the Mediterranean diet was applied, but a reverse coding system was used for dairy product intake.^13^ Thus, uncertainty remains about the role of dairy products in chronic disease outcomes. On the one hand, dairy products are presumed to be beneficial, as they are nutrient dense with large amounts of calcium, magnesium, vitamin A, vitamin D (when fortified), and high-quality protein.^14^ On the other hand, their contribution to saturated fat intake is seen as potentially detrimental to health outcomes.

Contrary to expectations and based on the strong focus on saturated fat as a risk factor for cardiometabolic disease, recent appraisal of the evidence has not been convincing for the effects of saturated fatty acids on such outcomes.^15^^,^^16^ Simultaneously, a dialogue has begun on whether the focus of dietary advice should move away from nutrients to a food-based approach.^17^ In light of these developments, it has become of great interest to investigate the potential role the intake of dairy products could have on cardiometabolic health. The focus of the present report is on the European Prospective Investigation into Cancer (EPIC) study’s contribution to furthering understanding of the association between the amount and type of dairy product consumption and the risk of developing incident type 2 diabetes.

In particular, EPIC investigators addressed 2 interlinked objectives in order to advance this field of inquiry. The first objective was to investigate the association between consumption of different amounts and types of dairy products and the development of incident type 2 diabetes; this was done using the heterogeneity of dietary exposures measured with food-frequency questionnaires (FFQs) across 8 European countries in the EPIC-InterAct study.^18^ The second objective was to investigate the association between dairy intake and diabetes using more detailed dietary information obtained from a prospective 7-day food diary in the UK–based EPIC Norfolk study.^19^

## RATIONALE, METHODS, AND FINDINGS FROM THE EPIC STUDY

### EPIC-InterAct study: intake of dairy products across 8 European countries

At the time the InterAct project was conducted,^20^ little research evidence was available from Europe on the association between dairy products and incidence of diabetes; only 3 studies had been published that, together, included fewer than 600 incident cases of type 2 diabetes, and 2 of those studies were restricted to men.^21–23^ The majority of past research had been conducted in the United States and Asia, where the intake of dairy products is generally lower than in Europe.^24^ In addition, there are differences in the nutritional composition of dairy products by location. Although the large variation across Europe in the intake levels of different types of dairy products had previously been described,^25^ an appraisal of the association between different types of dairy products with diabetes risk had not been undertaken in this population. Thus, it was timely and appropriate to undertake this analysis within InterAct.

Described in detail previously,^18^^,^^20^ the EPIC-InterAct study was a case-cohort study nested within 8 of the 10 countries participating in the EPIC study. A total of 340,234 EPIC participants were followed up between 1991 and 2007 for 3.99 million person-years; among them, the InterAct consortium partners ascertained and verified 12,403 incident cases of type 2 diabetes and randomly selected a subcohort of 16,835 individuals. After exclusions, the sample eligible for analysis included 10,694 diabetes cases and 13,780 subcohort participants, with 673 diabetes cases present in the subcohort, as per the design of the case-cohort study, which allowed for a small number of future incident cases to be included randomly within the subcohort. The statistical analysis took this design characteristic into account.^26^ Dietary intake was assessed by locally developed and validated semiquantitative FFQs.^27^ Intake of total dairy products was calculated as the sum of all dairy subtypes reported in the dietary questionnaires, with the exception of butter, which was not included. For the analysis of dairy subtypes, the study included intakes of milk, yogurt, thick fermented milk and cheese; a combined category of fermented dairy products comprised the sum of cheese, yogurt, and thick fermented milk.^18^ Statistical analysis used a modified Cox regression suitable for the case-cohort design.^26^ Country-specific hazard ratios (HRs) and 95% confidence intervals (CIs) for associations of quintiles of dairy products and dairy subtypes with incident type 2 diabetes were calculated, and a random-effects metaanalysis was performed to calculate a pooled HR. A series of statistical models was constructed that accounted for several relevant potential confounding factors including study center, age, and sex (model 1); plus body mass index (BMI), education level, smoking status, physical activity level, alcohol intake (model 2); plus total energy intake and energy-adjusted intakes of fruits, vegetables, red meat, processed meat, sugar-sweetened soft drinks, coffee, cereals, and cereal products (model 3). A number of sensitivity analyses and tests for interaction were prespecified.

Analyses found no significant association between diabetes and total dairy product intake or milk intake, but a higher combined intake of fermented dairy products (cheese, yogurt, and thick fermented milk) was inversely associated with diabetes (HR, 0.88; 95% CI, 0.78–0.99; *P* trend, 0.02) in adjusted analyses comparing extreme intake quintiles. In separate analyses for yogurt and thick fermented milk intake, there was an inverse association with diabetes incidence in model 1, which was rendered nonsignificant after further adjustment. For cheese intake, there was a significant inverse association with type 2 diabetes in model 1 (HR, 0.84; 95% CI, 0.74–0.95), but this was attenuated and became nonsignificant upon further adjustment for confounders, though an inverse trend remained across increasing quintiles of cheese intake (*P* linear trend, 0.01). These findings were robust to a range of sensitivity analyses, and there were no interactions between dairy product intake and each of sex, BMI, physical activity, or smoking habit and the risk of type 2 diabetes.

### EPIC-Norfolk study: intake of dairy products assessed using a prospective food diary

Though the EPIC-InterAct study provided the first large-scale evidence across Europe for the relationships of total and subtypes of dairy products and diabetes and contributed meaningfully to the research area, there were further unresolved issues. The issue of distinguishing between low-fat and high-fat dairy products could not be addressed in InterAct, while other studies that addressed this relied largely on participants’ self-report of preselected food items, indicating low-fat varieties with a variable degree of comprehensiveness.^8^^,^^10^ This was due to the fact that research thus far had predominantly used the FFQ, which is a commonly used dietary assessment tool in nutritional research because of its comparative ease of dietary data collection and its relatively lower cost to administer and analyze in large studies. Well-known limitations, however, include the restrictive preselected list of food items as well as the issues of errors due to misreporting based on the need to recall dietary information over the prior year. The EPIC-Norfolk study provided a unique opportunity to assess dietary intake of dairy products with a real-time 7-day food diary. This offered the advantages of being able to capture the intake of all food items consumed by participants, including dairy products as main ingredients in composite dishes. Food weights were estimated using photographs that represented portion sizes, household measures, and standard units.^28^ It also enabled the categorization of reported dairy product intakes into high- (or full-fat) and low-fat using 3.9% fat as a cutoff point, representing the fat content of whole milk in the United Kingdom. High-fat dairy included whole milk; all hard, processed, and soft cheese; full-fat, unripened cheese; cream; sour cream; crème fraiche; and butter. Low-fat dairy included all yogurt, semi-skimmed and skimmed milk, and low-fat unripened cheeses such as fromage frais and cottage cheese.^19^

The EPIC-Norfolk study is a population-based cohort study in Norfolk, United Kingdom, which recruited 25,639 men and women aged 40–79 years from lists of family physicians at baseline in 1993–1997.^29^ Participants have been followed up for incident events. The case ascertainment and verification exercise used multiple sources of information with record linkage to medical records and yielded 892 incident cases of type 2 diabetes through July 2006. The investigators assembled a nested case-cohort design. This included 4000 subcohort participants selected at random from the entire cohort. Due to the random nature of the subcohort, 143 of the future 892 type 2 diabetes cases were included within the subcohort, which the case-cohort design allows for in the analysis (as described above for the design of the EPIC-InterAct study). After exclusions, the final sample included 4127 participants (753 diabetes cases and 3502 subcohort participants, including 128 cases in the subcohort).^19^ With analyses that accounted for the case-cohort design,^26^ modified Cox regression models were used with comprehensive adjustments for confounding factors. Model 1 included age and sex; model 2 additionally included BMI, family history, smoking, alcohol intake, physical activity, social class, and education; and model 3 additionally included dietary factors (total energy intake and intake of fruits, vegetables, red meat, processed meat, fiber, and coffee).^19^ A number of sensitivity analyses and tests for interaction were included to test the robustness of the findings.

The higher consumption of low-fat fermented dairy products was associated with a lower risk of new-onset diabetes over 11 years compared with nonconsumption. Low-fat fermented dairy products consisted largely (87%) of yogurt but also included low-fat, unripened cheeses, e.g., fromage frais. In adjusted analyses, the HR for the association of low-fat, fermented dairy (highest compared with lowest tertile of intake) with incident diabetes was 0.76 (95% CI, 0.60–0.99; *P* trend, 0.049). For yogurt intake, the corresponding HR was 0.72 (95% CI, 0.55–0.95; *P* trend, 0.017). Other subtypes of dairy and total dairy, whether high fat or low fat, were not significantly associated with type 2 diabetes risk.

### Interpretation of findings from the EPIC-InterAct and EPIC-Norfolk studies

Despite the differences in the detail of dietary assessment used, with country-/center-specific FFQs in the EPIC-InterAct study and the 7-day food diary in the EPIC-Norfolk study, the overall findings were remarkably consistent. These findings suggested that the consumption of dairy subtypes, particularly of the fermented variety, rather than all dairy, may be beneficial for the prevention of diabetes, highlighting the relevance of food group subtypes for public health messages.

Overall, observational evidence for the connection between dairy intake and diabetes has been summarized to date in 4 metaanalyses.^7–10^ Two of them included the results from the EPIC-InterAct study,^9^^,^^10^ but none included the findings from the EPIC-Norfolk study, as these were unavailable at the time the metaanalyses were published. Within the metaanalyses, the pooled analyses showed an inverse association with total dairy intake, a finding not observed in the EPIC-based analyses, which may be due to the association with some, but not all, dairy subtypes.

While the analyses from the EPIC-InterAct and EPIC-Norfolk studies had several strengths, including the large sample size and number of cases included, the prospective study design, and the comprehensive adjustment for several relevant confounding factors, some of the limitations of nutritional epidemiology remain. The issue of misreporting based on recalled dietary intake with the FFQ was minimized by the use of the 7-day food diary (which records intake in real time) in the EPIC-Norfolk study. However, without repeat dietary assessment, both studies could not account for change in dietary habits over time. The issue of potential residual confounding is a possibility in both studies as the confounding factors may be measured with error or unknown confounders may remain unaccounted for.

A cause-and-effect relationship, for which a randomized trial would provide the highest form of evidence, cannot be established. However, in reality, such a trial is unlikely to be feasible for a dietary intervention for a “hard” endpoint such as type 2 diabetes, which would require participants to adhere to particular diets for several years in order to allow enough time for onset of the disease. Alternatively, criteria such as those proposed by Hill^30^ can be used to appraise the likelihood of causal inference, including strength of association, consistency, repeatability, specificity, temporality, dose response, and biological plausibility. Regarding the final point, though the mechanisms of association between intakes of subtypes of dairy products and incidence of type 2 diabetes are not well understood, several possibilities exist. Potential mechanisms through which dairy products may generally exert beneficial effects include the many vitamins and minerals included in these products, such as calcium, vitamin D (in fortified dairy), and magnesium as well as high-quality protein. More specifically, fermented dairy products may have additional benefits through probiotic bacteria and menaquinones, as previously discussed.^18^^,^^19^ Whether individual saturated fatty acids from dairy products also play a role in the etiology of type 2 diabetes is of interest but has been little researched. The EPIC-InterAct study provided an opportunity to investigate this issue.

### EPIC-InterAct study: rationale and findings for objectively measured saturated fatty acids

Fatty acids are the building blocks of fat, and reducing the consumption of saturated fatty acids (SFAs) to below 10% or even below 7% of total energy intake has been deeply embedded in dietary guidelines.^11^^,^^31^ The focus on dietary SFA reduction was based on cardioprotection related to the direct association between SFA intake and total- and LDL-cholesterol levels, the latter being an established risk factor for coronary heart disease. SFA intake has also been considered a risk factor for insulin resistance and diabetes.^32^^,^^33^ However, a recent appraisal of the evidence highlighted the equivocal nature of the previous conclusions about SFA intake for both cardiovascular disease and diabetes.^15^ For diabetes, neither observational evidence nor trial evidence supported an adverse effect of high SFA intake on risk of type 2 diabetes.^15^ Indeed, the Women’s Health Initiative Diet Modification trial suggested no benefit of a reduction in SFA intake on the incidence of type 2 diabetes.^34^ A further issue is that within the SFA/metabolic disease paradigm there is accumulating incongruous evidence that dairy products, which are typically high in SFA content, are inversely associated with incident type 2 diabetes.^7–10^^,^^18^^,^^19^

In identifying the reasons for some of the observed discrepancies, it is important to acknowledge that previous research on SFA and diabetes focused on total SFA intake, without distinguishing between SFAs of different carbon chain lengths, which can have important differences in biological action. This, in turn, has been the result of past nutritional research that relied on dietary assessment based on self-report from questionnaires, which did not readily permit the examination of SFA of different carbon chain lengths. In contrast, the objective measurement of SFAs of different carbon chain lengths in blood factions enables the assessment of individual SFAs.^35^ While there are complexities of uncertainty about the extent to which circulating individual SFAs represent diet vs endogenous processes, it is of great interest to investigate the association between individual SFAs of different carbon chain lengths and incident type 2 diabetes in order to inform this field of inquiry. Past evidence is restricted to a handful of studies limited in sample size, number of diabetes cases, and the varying number of SFAs assessed using different methods.^36–42^

Thus, the aim was to investigate the prospective association between objectively measured individual SFAs in the plasma phospholipid fraction and incident type 2 diabetes using the advantages of the EPIC-InterAct study, including variation in SFA levels across 8 European countries.^43^

Described in detail previously,^43^ for this investigation, a profile of 37 fatty acids in the plasma phospholipid fraction was measured using gas chromatography.^44^ Each fatty acid was expressed in relative units as the percentage of total phospholipid fatty acids (mol%). Nine SFAs of different carbon chain lengths and with relative concentrations higher than 0.05% were included in the analyses, of which 15:0 and 17:0 were the 2 SFAs considered derived from dietary dairy fat.^35^^,^^45^^,^^46^ In analyses that accounted for a range of potential confounders such as sociodemographics, obesity, and lifestyle factors, including diet and energy intake, these odd-chain SFAs were associated inversely with incident diabetes. Per 1 standard deviation difference in SFA, the HR for 15:0 (pentadecanoic acid) was 0.79 (95% CI, 0.73–0.85), and the HR for 17:0 (heptadecanoic acid) was 0.67 (95% CI, 0.63–0.71). In contrast, the even-chain SFAs were positively associated (14:0 [myristic acid] HR 1.15 [95% CI, 1.09–1.22], 16:0 [palmitic acid] HR 1.26 [95% CI, 1.15–1.37], and 18:0 [stearic acid] 1.06 [95% CI, 1.00–1.13]). When comparing quintiles of the SFA distribution for the odd-chain SFAs, the adjusted HR comparing the top with the bottom quintile of 15:0 was 0.46 (95% CI, 0.37–0.56; *P* trend, < 0.0001) and for 17:0 it was 0.24 (95% CI, 0.20–0.30; *P* trend, < 0.0001). Conversely, for the even-chain SFAs, the corresponding HRs were 1.64 (95% CI, 1.47–1.83) for 14:0, 1.75 (95% CI, 1.35–2.27) for 16:0, and 1.75 (95% CI, 1.46–2.09) for 18:0 (*P* trend across quintiles was < 0.0001 for all even-chain SFAs). Interpretation of the findings for even-chain SFAs is complex because these circulating SFAs are mainly derived from hepatic endogenous synthesis (de novo lipogenesis), stimulated by intakes of carbohydrates and alcohol.^35^^,^^47–49^ However, the findings for odd-chain SFAs (15:0 and 17:0) can be understood in terms of their exogenous source from dairy fat.^35^^,^^45^^,^^46^

### Overall contribution from the EPIC study and its implications

With the EPIC-InterAct study across 8 European countries and the EPIC-Norfolk study in the United Kingdom, large-scale and robust evidence has been generated among European populations on the association between the consumption of dairy products and the incidence of type 2 diabetes. There was a remarkable consistency of findings for fermented dairy products being inversely associated with diabetes across the 2 dietary instruments (FFQ in the EPIC-InterAct study and the 7-day food diary in the EPIC-Norfolk study). The EPIC-Norfolk study allowed greater differentiation by fat content status, i.e., low-fat fermented dairy products (including yogurt, where all yogurt was low-fat by virtue of <3.9% fat content) were inversely associated with diabetes risk. Moreover, the measurement of individual circulating SFAs in the InterAct study enabled the world’s largest appraisal of the association of SFAs of different carbon chain lengths with the risk of type 2 diabetes. This is an important step toward recognizing that SFAs are not a single homogenous group and that differences exist between the differential health effects of subtypes of blood SFAs. The question of whether 15:0 and 17:0, presumed derived from dairy fat, have direct physiological effects on the development of diabetes or whether they are markers of other components in dairy is currently unclear and should be the subject of further research, together with gaining a better understanding of the extent to which the content of these odd-chain SFAs varies by type of dairy product. The implication, however, of the EPIC-InterAct study’s blood fatty acid biomarker findings is that it informs the recognition that it is not enough to provide public health messages about overall saturated fat intake, but that more nuanced messages acknowledging the food sources of different types of SFAs are required.

Taken together, the findings from the EPIC study (EPIC-InterAct and EPIC-Norfolk) indicate that a public health focus solely on nutrients (e.g., SFAs) may be misplaced, and what is required is consideration of the food sources associated with those nutrients. For instance, both meat and dairy products are rich in total fat and SFAs, but their association with type 2 diabetes is in opposite directions: a positive association has been observed between red and processed meat intake and diabetes risk,^50–54^ while there is now consistent evidence from EPIC^18^^,^^19^ and elsewhere^7–10^ for an inverse association between the consumption of specific types of dairy products and incident diabetes.

### Findings in context and future directions

Considerable progress has been made through the EPIC study and other studies that have advanced understanding of the relationship between dairy consumption and development of type 2 diabetes. However, it is important to note that dairy products should be consumed within an overall healthy diet. Moreover, healthy diets should be complemented with other healthy lifestyle factors, such as taking part in regular physical activity, maintaining a healthy weight, and not smoking, to provide greater potential for the prevention of type 2 diabetes and other chronic diseases.

More research on dairy products and health is warranted because there are still unanswered questions. A nonexhaustive list of currently unresolved issues is outlined in Box 1. Research continues in order to address some of these unresolved issues, and a concerted effort by the scientific community will be needed to tie together the different strands of evidence that range from observational to experimental. A recent review has summarized much of the evidence thus far, including that from randomized clinical trials showing the effects of dairy intake on intermediate markers of cardiometabolic risk,^55^ but much more research is still needed. Greater collaboration amongst different disciplines is also required to undertake collaborative research that spans nutritional epidemiology and dietary public health, as well as the study of physiological processes and biological mechanisms that underpin associations between dairy consumption and health outcomes.

Box 1 Sample of unanswered research questions regarding dairy products and health.

- If there is a cause–effect relationship between dairy product consumption and type 2 diabetes, does dairy intake exert direct effects on insulin sensitivity or are the effects on diabetes risk exerted through changes in weight or obesity?
- Which components of dairy products exert the health effects, including specific fatty acids, protein, minerals, vitamins, and constituents associated with fermentation? What are the roles of milk sugars in dairy products? What are the mechanisms by which the components of dairy products exert health effects?
- To what extent are there differences in composition (e.g., of SFA content) and health effects by the type of dairy product, such as full-fat, low-fat or reduced-fat or fermented/nonfermented dairy, and are these dependent on cattle-feed? More specifically, how do different types of dairy products vary in their content of 15:0 and 17:0?
- What is the net effect on cardiometabolic risk of consuming reduced-fat dairy products that have added sugars?
- As sources of animal-derived protein and fat, how do dairy and meat products compare in their relative effects on cardiometabolic health, and is there a difference in meat products from ruminants vs other animals?
- Do the odd-chain SFAs such as 15:0 and 17:0 have direct physiological benefits or are they merely correlates of other beneficial substances in dairy products?
- What is the effect of fatty acids in dairy products (other than SFAs) on cardiometabolic risk?
- Are there healthy and less healthy dairy options? Should butter be included within the definition of “dairy” or kept separate?
- To what extent is the consumption of dairy products a marker of overall (healthier or unhealthier) diets and (healthier or unhealthier) lifestyles?
- What are the facilitators and barriers to dairy product consumption?

## CONCLUSION

Efforts to understand the mechanisms of association and to investigate potential cause–effect relationships between dairy consumption and health outcomes are ongoing, but the collective epidemiological findings thus far suggest that specific types of dairy products, particularly fermented dairy products including yogurt, may help prevent type 2 diabetes within overall healthy lifestyles. Such findings highlight the importance of considering food group subtypes (e.g., fermented dairy products such as yogurt), rather than overall food group categories (e.g., dairy products), when examining the role of diet in the prevention of chronic diseases.
